# Protectors or Traitors: The Roles of PON2 and PON3 in Atherosclerosis and Cancer

**DOI:** 10.1155/2012/342806

**Published:** 2012-05-13

**Authors:** Ines Witte, Ulrich Foerstermann, Asokan Devarajan, Srinivasa T. Reddy, Sven Horke

**Affiliations:** ^1^Department of Pharmacology, University Medical Center of the Johannes-Gutenberg University Mainz, Obere Zahlbacher Street 67, 55131 Mainz, Germany; ^2^Department of Medicine, University of California, Los Angeles, CA 90095, USA; ^3^Department of Molecular and Medical Pharmacology, University of California, Los Angeles, CA 90095, USA

## Abstract

Cancer and atherosclerosis are major causes of death in western societies. Deregulated cell death is common to both diseases, with significant contribution of inflammatory processes and oxidative stress. These two form a vicious cycle and regulate cell death pathways in either direction. This raises interest in antioxidative systems. The human enzymes paraoxonase-2 (PON2) and PON3 are intracellular enzymes with established antioxidative effects and protective functions against atherosclerosis. Underlying molecular mechanisms, however, remained elusive until recently. Novel findings revealed that both enzymes locate to mitochondrial membranes where they interact with coenzyme Q10 and diminish oxidative stress. As a result, ROS-triggered mitochondrial apoptosis and cell death are reduced. From a cardiovascular standpoint, this is beneficial given that enhanced loss of vascular cells and macrophage death forms the basis for atherosclerotic plaque development. However, the same function has now been shown to raise chemotherapeutic resistance in several cancer cells. Intriguingly, PON2 as well as PON3 are frequently found upregulated in tumor samples. Here we review studies reporting PON2/PON3 deregulations in cancer, summarize most recent findings on their anti-oxidative and antiapoptotic mechanisms, and discuss how this could be used in putative future therapies to target atherosclerosis and cancer.

## 1. Introduction

Most studies in the field of paraoxonases (PONs) deal with cardiovascular diseases, such as atherosclerosis and diabetes, where PONs exert protective functions in cell culture as well as animal studies. It has been anticipated that the known antioxidative functions of PONs, including PON2 and PON3, were central to their effects although underlying molecular mechanisms remained obscure. However, recent findings caused a significant progress in this field because molecular pathways of PON2 and PON3 functions have been largely revealed. Moreover, the result of the cell-protective function were shown to play a vital role in survival and stress resistance of cancer cells, along with the finding that numerous tumors overexpressed these enzymes. There, PON2 and PON3 appear to increase chemotherapeutic resistance and favor cell survival. In this review, we summarize the most recent findings and discuss the role of PON2/PON3 in atherosclerosis and cancer. A future perspective gives an outlook on how PONs may be targets of novel therapeutic approaches.

## 2. Altered Expression Levels of Paraoxonase Enzymes in Cancer

It is established that oxidative stress from mitochondria plays an important role in apoptosis and also leads to premature aging and cancer. There is growing scientific consensus that antioxidants or proteins with antioxidative functions, such as paraoxonases, can lower the incidence of, for example, cardiovascular and neurodegenerative diseases. On the other hand, recent studies have shown that various types of cancer obviously take advantage of this protection by enhanced expression of the antioxidative paraoxonase proteins. In the following section, we give an overview of studies that assessed expression of PON1, PON2, or PON3 in various cancers, with the majority of studies seemingly reporting a deregulation of these proteins.

PON1 levels and activity are lower in many inflammatory and oxidative stress-associated diseases [1]. Also, serum PON1 and arylesterase activities were reduced in patients with epithelial ovarian cancer [2] and lung cancer [3]. Uyar et al. found that Q allele of PON1 was more frequent in renal cancer patients [4], and Antognelli at al. reported that certain PON1 genotypes were prone to increased risk of prostate cancer [5]. More recently, the presence of the variant alleles of the Q192R and L55M SNPs of PON1, both of which result in an amino acid replacement that alters PON1 activity, were found associated with a 18–29% increased risk of aggressive prostate cancer [6]. These studies clearly demonstrate a link between PON1 and cancer etiology; however, PON1 is not the scope of this review. We will focus on the role of PON2 and PON3 in cancer based on recent discoveries on the mechanism of action of these proteins in proliferation and apoptosis. 

 Research on paraoxonases is a relatively young field, and still much of our understanding comes from findings related to PON1. Back in 1999, our knowledge about PON2 and PON3 was extremely limited although few studies emerged that reported genetic associations with metabolic diseases [7]. There are two common single nucleotide polymorphisms (SNPs) in PON2—G148A and C311S—that have been associated with disease phenotypes. In essence, an association between these SNPs and several diseases was demonstrated. For PON2-G/A148 this is true, for instance, for higher plasma glucose [8], higher plasma HDL cholesterol [9], and lower plasma LDL cholesterol [10]. With respect to S/C311, Stoltz et al. reported that this mutation determines the lactonase activity of PON2 which links to hydrolysis of important bacterial virulence factors [11]. However, a subsequent study from our lab did not confirm this finding [12]. The impact of PON2-S/C311 on lactone hydrolysis thus merits further investigation. This may similarly apply for its role in coronary heart disease, where at least one study reported an association [13] that was not found in a subsequent meta-analysis [14].

Despite the established and prevailing role of paraoxonases in cardiovascular diseases and relevant parameters, more recent studies revealed an emerging association of PONs with cancer. For example, microarray studies observed an overexpression of PON2 in some solid tumors like hepatocellular carcinoma, prostate carcinoma [15, 16], and several others, which are illustrated in Table 1. Additionally, in various leukemia gene expression profiling studies, an upregulation of PON2 could be demonstrated; an example is pediatric acute lymphoblastic leukemia (ALL) [17]. Importantly, a subsequent study identified PON2 as member of a very small group of upregulated genes that characterized pediatric ALL patients with very poor outcome prognosis [18]. In another form of leukemia, chronic myeloid leukemia (CML), PON2 was also identified in an outcome-specific gene expression signature of primary imatinib-resistant patients [19]. Moreover, a marked overexpression of PON2 was observed in lymphocytes infected with T-cell leukemia virus [20].

In contrast to PON2, there are fewer studies for PON3 with the tendency of more diverse results (see Table 2). For instance, a downregulation was demonstrated in a meta-analysis of expression profiles in hepatocellular carcinomas (HCC) [21] and in ovarian serous papillary carcinomas (OSPCs) [22] shown by oligonucleotide microarrays. However, there are various other such analyses, which showed altered expression of PON3 (up as well as downregulated) in different types of cancers. For overview, consult the Gene Expression Atlas found at http://www.ebi.ac.uk/gxa/. In general, it should be noted that these association studies show no direct proof for a physiological relevance of these proteins in cancer, nor do such studies give any clues about their functions and mechanisms.

In addition to the listed microarray data, our very recent analyses showed that the PON2 level is increased in some tumors at the protein level (Table 1). We showed a moderate PON2 overexpression in pancreas, liver, kidney, and lung tumors and an over 10-fold upregulation of PON2 in thymus tumors and non-Hodgkins lymphomas [23]. Assessment of PON2 protein levels is not feasible in hundreds of cancer samples. Therefore, we previously used cDNA arrays, developed for differential gene expression analysis and validation of hundreds of different human tissues. We showed that PON2 is ~2–4-fold overexpressed in the tumors from urinary bladder, liver, kidney, lymphoid tissues, and endometrium/uterus in comparison to normal tissue [23], which are in accordance with western blot analyses. Despite some other tissues, where no increase in the expression level was observed, human tumors of the thyroid gland, testis, prostate, and pancreas showed a slight upregulation of PON2 (Table 1).

Using the same cDNA arrays as for PON2, our group showed a considerably increased PON3 expression in all tested cancer types, except cervix [24]. Remarkably, the intensity of PON3 overexpression was markedly enhanced compared to that of PON2. In this array over 10-fold upregulation of PON3 in tumors from endometrium/uterus and stomach was shown and over 3-fold induction in samples from pancreas, urinary bladder, thyroid, prostate, pancreas, liver testis, and lung cancers. These results could be verified with another matched array particularly for lung cancer (normal versus diseased samples from the same patient). But in contrast to PON2, PON3 expression appears to be largely restricted to cells derived from solid tumors [24]. One reason for the high expression level of PON3 in cancer tissue is certainly the low basal expression level of PON3 in healthy tissues but may nevertheless suggest a role for PON3 in cell death escape.

**Figure 1:**
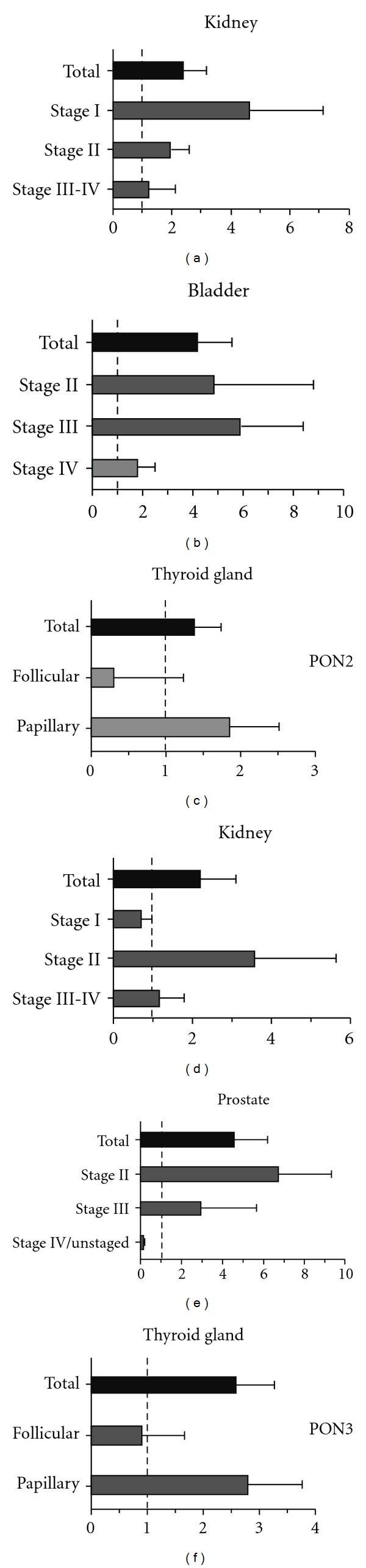
PON2 and PON3 are found overexpressed in early rather than late stages of tumors. Indicated cancer tissues were analyzed for PON2/PON3 cDNA levels (normalized to GAPDH) relative to healthy controls. Values were taken from recently performed arrays [23, 24].

 An interesting phenomenon is obvious upon closer inspection of the array data. A tumor subtype and stage-specific analysis revealed that both PON2 and PON3 are upregulated rather in the early stages and some subtypes of cancer, whereas the expression in the late stages of the tumor seems to be declining (see Figure 1). This could indicate that, especially in the early stages of tumor formation, the antioxidative and antiapoptotic function of PON2 and PON3 is important and beneficial as it helps generating the platform for malignant transformation. This could represent a potential approach of innovative therapies trying to normalize the otherwise overexpressed PONs.

 A first direct hint to this theory came from our recent study demonstrating that PON2 increased chemoresistance in leukemic cells [23], which is in line with genetic association studies where PON2 upregulation was associated with imatinib resistance in CML patients [19] and poor prognosis in cohorts of pediatric ALL [17, 18]. In support of the hypothesis, the same study [23] revealed that knockdown of endogenous PON2 caused spontaneous apoptosis of several human cancer cell lines—an intriguing but somewhat unexpected finding given the viability of PON2-deficient mice (the residual PON2 expression in these mice [25] may be comparable to efficient cell culture RNAi experiments).

An exciting question is how tumors achieve an increase in PON2 and/or PON3 expression, and this should be a major goal of future studies. Certainly there is no general answer to this question. Most likely, underlying mechanisms are individual for each given tumor. One simple explanation could be that, in some tissues, for example, papillary renal cell kidney carcinoma or prostate adenocarcinoma, chromosome 7, which contains the PON cluster, is amplified [16]. Another reason might be that the regulation depends on several signaling pathways, which are linked to reactive oxygen species and cancer, for example, PPAR-*γ*, AP-1, *β*-catenin/Wnt, NF-*κ*B, HIF-1*α*, PI3K, and Nrf2 [26]. In accordance, earlier studies showed that PON2 expression is enhanced by oxidative stress [27], PI3K/PDGFR, PPAR*γ*, and NADPH oxidase activation as well as by AP-1 activation [28, 29]. The urokinase plasminogen activator (uPA) system may also be relevant, as this is increased in numerous cancers and upregulates PON2 [29].

A point of interest is why some tumors upregulate PON2 or PON3. One of the hallmarks of cancer is resistance to cell death [30]. It has been found that paraoxonases 2 and 3 provide a protection against mitochondrial cell death signaling [23, 24]. Their overexpression lowered susceptibility to different chemotherapeutics (e.g., imatinib, doxorubicin, and staurosporine) in cell culture models via diminishing proapoptotic mitochondrial O_2_^−^ formation. It is established that oxidative stress and chronic inflammation are closely linked to cell death and cancer [26]. Therefore, it appears conceivable that tumors take advantage of the antioxidative function of PON2/PON3 to escape cell death.

## 3. The Antioxidative Mechanisms of PON2/PON3

Inflammation and oxidative stress contribute to the etiology of almost every known disease. Reactive oxygen species generated by enzymatic and nonenzymatic systems modify lipids and sterols, producing oxidized lipids and oxidized sterols that, if unchecked, produce a vicious cycle of undesirable inflammation and more oxidative stress. Atherosclerosis is a chronic inflammatory disease characterized by the focal accumulation of numerous cells, lipids, and extracellular matrices in the intima of arteries. Although reduced levels of high density lipoprotein (HDL) and elevated levels of low density lipoprotein (LDL) cholesterol are accepted risk factors for this disease, atherogenesis cannot solely be explained by cholesterol or lipid deposition in the arterial wall. Accumulating evidence suggests that oxidative stress plays a fundamental role in atherosclerosis. In particular, the oxidation theory for atherosclerosis proposes that LDL is a major target of oxidation and is involved in both the initiation as well as progression of atherosclerosis [31].

Although there has been a focus on PON1 due to its association with HDL, a number of studies demonstrated that PON2 and PON3 protect cells and tissues from oxidative stress by reducing reactive oxygen species [1, 25, 32–37]. PON2 and PON3 can inhibit LDL oxidation and enhance the antioxidant properties and cholesterol efflux capacity of HDL even though they are not readily found on the lipoproteins [1, 25, 32–37]. Moreover, in animal models, both PON2 and PON3 have been shown to abrogate the development of atherosclerosis [25, 35, 38]. These preclinical studies clearly demonstrated that PON2 and PON3 (similar to PON1) are (a) anti-atherogenic and (b) targets for therapy. However, to date, the physiological substrates and roles for PON2 and PON3 have not been elucidated, which similarly applies to PON1.

**Figure 2:**
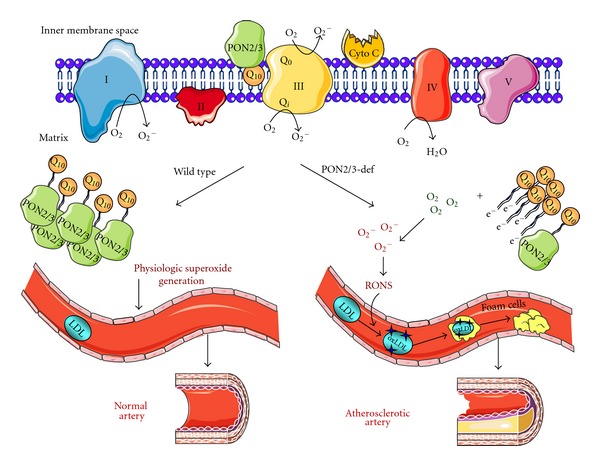
Schematic presentation of the suggested antioxidative mechanism of PON2 and PON3. A current model for the role of PON2/3 in the development of atherosclerosis. Ubisemiquinone is released from ETC in the mitochondria during Q cycle. *Right.* In the absence of PON2/3, ubisemiquinone donates electron to molecular oxygen to form superoxide; superoxide generates other reactive oxygen/nitrogen species (RONS), which oxidize LDL to form oxLDL; macrophages engulf oxLDL to form foam cells; foam cells attach to the arterial wall and subsequently develop into atherosclerotic lesions. *Left.* In the presence of PON2/3 (in wild type mice), ubisemiquinone binds to PON2/3. The binding of PON2/3 and ubisemiquinone prevents superoxide generation thereby preventing the development of atherosclerosis. Note: it is currently unknown if PON2/3 face the matrix side of the inner mitochondrial membrane or the one directed towards the innermembrane space; also, the stoichiometry of PON2/3 versus Q10 is unknown. Abbreviations: I-NADH: ubiquinone oxidoreductase, II: succinate coenzyme Q reductase, III: ubiquinol cytochrome coxidoreductase, IV: cytochrome c oxidase, V-ATP synthase, Cyto c cytochrome c, Q10-coenzyme Q10.

Recent studies suggest that PON2 [12, 38, 39] and PON3 [24] modulate the levels of reactive species in cells and in animal models demonstrating for the first time a physiological molecular link between PON proteins and oxidative stress. Based on the earlier result that PON2 was found in subcellular mitochondrial fractions [40], Altenhöfer et al. demonstrated that PON2 prevents the ubisemiquinone-mediated mitochondrial superoxide generation and apoptosis independent of its lactonase activity [12]. During Q cycle, unstable intermediate ubisemiquinone (coenzyme Q_10_ [CoQ_10_^−^]) can donate electron to molecular oxygen (instead of cytochrome c) leading to superoxide production and reduced ETC activity [41–43]. Devarajan et al. reported that (a) PON2 is present in the inner mitochondrial membrane (IMM), and (b) binds with high affinity to coenzyme Q10 (CoQ10), an important component of the ETC [38]. Steady-state concentrations of ubisemiquinone are increased in the IMM resulting in superoxide formation when treated with inhibitors of ETC, antimycin, or rotenone [43]. Devarajan et al. demonstrated that overexpression of PON2 reduces superoxide levels induced by either antimycin or rotenone suggesting that PON2 sequesters ubisemiquinone. Moreover, PON2-deficient mice harbour reduced ETC complex I + III activities, oxygen consumption, ATP levels, and enhanced mitochondrial oxidative stress further suggesting that PON2 maintains the respiratory chain by promoting the sequestration of the unstable reactive intermediate ubisemiquinone, thereby preventing the superoxide production. Supporting our hypothesis, previously, it has been shown that mitochondrial superoxide is inversely related to the amount of CoQ10 bound to membrane proteins [44]. Similar to PON2, Schweikert et al. have demonstrated that PON3 is also localized to mitochondria, protects against mitochondrial oxidative stress, and demonstrated that Q10 is associated with purified PON3-GFP protein [24]. This illustrates that the antiatherogenic effects of PON2/3 are, in part, mediated by their role in mitochondrial function (Figure 2). Since increased production of reactive oxygen species (ROS) as a result of mitochondrial dysfunction play a role in the development of many inflammatory diseases including atherosclerosis, the recent data on PON2 and PON3 provide a mechanistic direction for the scores of epidemiological studies that show a link between PON proteins and numerous inflammatory diseases including Type II diabetics and cancer.

Atherosclerosis and insulin resistance are multifactorial diseases that are commonly associated with dyslipidemia, oxidative stress, obesity, hypertension, and chronic inflammation. The liver is not only the primary site of lipid metabolism, but is a major site for glucose uptake, production, and storage. Its role in glucose metabolism is strongly influenced by systemic as well as local oxidative and inflammatory stimuli [45, 46], which in turn influences whole-body insulin responsiveness [47]. Hepatic glucose metabolism is strongly influenced by oxidative stress and proinflammatory stimuli. Given the elevated oxidative stress levels and abnormal lipid metabolism reported previously in PON2-deficient mice [25, 38], Bourquard et al. hypothesized that atherosclerosis may be accompanied by impaired hepatic insulin signaling and showed that PON2 deficiency is associated with inhibitory insulin-mediated phosphorylation of hepatic insulin receptor substrate-1 (IRS-1) [39]. Factors secreted from activated macrophage cultures derived from PON2-deficient mice are sufficient to modulate insulin signaling in cultured hepatocytes in a manner similar to that observed *in vivo* [39]. It was further demonstrated that modulation of hepatic insulin sensitivity by PON2 is mediated by a shift in the balance of NO and ONOO^−^ (peroxynitrite) formation. These studies show that PON2 plays an important role in insulin sensitivity by its ability to modulate reactive species most likely as a result of PON2's association with mitochondrial function.

Oxidative stress has long been associated with the pathophysiology of cancer. In particular, enhanced ROS formation increases DNA damage, genome instability, and cell proliferation especially during cancer initiation. On the other hand, oxidative stress also counteracts tumorigenesis, as it induces senescence and drives apoptosis and other cell death pathways [48]. The precise spatiotemporal control of ROS generation is therefore a critical regulator of cell survival and death, for instance since overwhelming mitochondrial oxidative stress exerts apoptotic rather than protumorigenic functions. Nevertheless, reactive oxygen species may be conducive to the vitality of cancer cells and drive signaling transduction pathways, which lead to activation of redox-sensitive transcription factors and genes involved in cancer cell growth, proliferation, and survival [26]. In conclusion, PON2 and PON3 reduce oxidative stress and inflammation and thus act as central regulators of diseases, including cancer and atherosclerosis.

## 4. Paraoxonases and the Regulation of Cell Death

The antioxidative effects of PON2 and PON3 were reported long ago, but underlying mechanisms were uncovered just recently [12, 24, 38]. This similarly applies to the cell death-reducing activity of PON2, where discovery [40] and mechanistic realization [23] were separated by years. Based on the latest knowledge, these enzymes modulate execution of the apoptotic program. In this chapter, we review their involvement in apoptosis and discuss their putative functions in other cell death pathways.

Tumor cells evolve a plethora of strategies to resist cell death with the intrinsic apoptotic program being implicated as a major barrier to cancer formation. Execution of intrinsic cell death is mainly controlled by the balance of pro- and antiapoptotic Bcl-2 protein family members [30, 49], because they regulate mitochondrial pore opening and cytochrome C release. Importantly, it also requires intramitochondrial redox signaling to liberate cytochrome C from its membrane attaching molecule, cardiolipin [50, 51]. In fact, this is a two-step process because neither mitochondrial membrane permeabilization alone nor redox-triggered disruption of the cytochrome C/cardiolipin interaction sufficiently activates the cascade. Recent studies revealed that PON2 and PON3, due to interaction with coenzyme Q10, diminish O_2_^−^ release on either side of the inner mitochondrial membrane [23, 24]. This results in both lowered cardiolipin peroxidation and cytochrome C release, providing a marked resistance against apoptosis. Thus, if a cancer cell needs to escape from mitochondrial redox-dependent cell death, it appears beneficial to increase PON2 or PON3 expression. In accordance, both enzymes protected against a range of chemotherapeutics when overexpressed [23, 24]. In contrast, receptor-mediated apoptosis was unchanged, at least in type-I cells, where stimulation with TRAIL or TNF-*α* directly activated caspases 8 and 3. This may be different for type-II cells, which involve mitochondrial actions.

**Figure 3:**
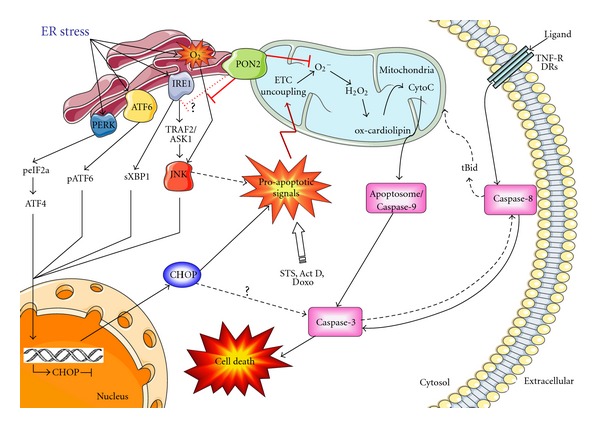
Schematic presentation of the suggested antiapoptotic mechanism of PON2 and PON3. Its ability to prevent mitochondrial O_2_^−^ formation impacts on both ER stress-induced pathways (via acting on JNK and CHOP) as well as mitochondrial proapoptotic signaling such as cardiolipin peroxidation and cytochrome C release. See text for details. From our current understanding, PON2 is functionally interchangeable with PON3.

Another important stress and cell death pathway is the unfolded protein response (UPR) as a result of insurmountable ER stress [52]. Both PON2 and PON3 protected against UPR-mediated apoptosis in a similar manner, that is, by negative modulation of JNK signaling, CHOP induction, and subsequent caspase activation [12, 23, 24]. Canonical UPR signaling (via ATF6, XBP1, or p-eIF2a) was unchanged, at least by PON2, so their precise mechanisms of protection remain uncertain. Future studies must reveal if PON2/PON3 act just through their mitochondrial effects or if they modulate signaling from IRE1 to TRAF2/ASK1, from the ER to mitochondria or local ROS/Ca^2+^ responses and how they reduce JNK phosphorylation. Interestingly, PON2 overexpression was induced by ER stress and protected against UPR-triggered cell death, but this was lost upon major disturbances of Ca^2+^—homeostasis, presumably by calpain-dependent PON2 degradation [53]. Our current studies suggest that this similarly applies to PON3 ([54] this issue and data not shown). The postulated functions of PON2 and PON3 in apoptosis and ER stress-induced cell death are summarized in Figure 3.

A vital physiologic response that regulates cellular metabolism and survival is autophagy. This pathway operates at low basal levels but can be markedly increased under specific stress conditions. It enables breakdown of macro-molecular structures and organelles to allow recycling of catabolites. Therefore, autophagy may alleviate nutrient limitation as experienced by many cancer cells. However, autophagy has opposing effects on different tumor cells and may cause survival of one but death of the other [55]. Whether paraoxonases modulate this pathway is unknown and no interaction with Bcl-2 family/autophagy-related proteins has been reported. On the other hand, oxidative stress is mutually linked with autophagy, and there it plays an important role in cancer therapy resistance and tumor progression. The connection between ROS and autophagy is illustrated, for instance, by TNF*α*-induced signaling in sarcoma cells [56], or by the autophagy-relevant factor Atg4 whose delipidating activity is sensitive to mitochondrial H_2_O_2_ production [57] (see [58] for a detailed overview of this topic). Paraoxonases hence could have a profound impact on autophagy due to their central redox effects. Because autophagy by ROS can serve as rescue pathway but may also initiate autophagic cell death, it requires more in-depth evaluation including the origin and targets of ROS. In a similar manner, this may also be true for necroptosis (or necrotic cell death), which contrasts with the chemical- or injury-triggered necrosis and represents another, RIP1 kinase-dependent programmed cell death pathway. Necroptosis is of relevance, for example, for damages resulting from ischemia-reperfusion, such as stroke or myocardial infarction. Moreover, necrotic cell death may paradoxically be even beneficial to neoplasias as this form of cell death attracts tumor-promoting inflammatory cells [30]. TNF-induced necroptosis has been shown to generate complex-I-mediated ROS in mitochondria, which is crucial to this process and accounts for ultrastructural changes observed in such cells [59]. Because PON2 as well as PON3 were able to reduce superoxide released from mitochondrial complex-I [12, 24, 38], it would hence be a promising endeavor to test PONs in TNF-induced necroptosis.

Another hallmark of tumor cells is the reprogramming of glucose metabolism in order to provide efficient fueling of the high energy demand associated with rapid cancer growth. For the most part, this is manifested as a switch to (aerobic) glycolysis but also involves two different cancer cell subpopulations—one using glucose and a second set consuming lactate produced by the former (see [30] and references therein). How overexpressed PONs could play a role in this system has not been explored, and speculations can only be extrapolated from the PON2-deficient mice. Intriguingly, mitochondria from PON2 deficient mice produced less ATP, had impaired complex-I and -III activities, and showed enhanced oxidative stress and consumed less oxygen, resulting in an overall exhausted mitochondrial function [38]. Thus, if the opposite was true for PON2 overexpressing cells, this would ensure mitochondrial functionality and could support the energy efficacy of tumor cells.

Despite a role of paraoxonases directly in cancer cells, it could also be interesting to scan for other near-by functions. Cancer progression is determined by intracellular changes in the malignant cell itself, but also modulated by surrounding stromal cells in the tumor microenvironment. It is composed of leukocyte infiltrates consisting, for example, of endothelial cells, mast cells, T cells, and tumor-associated macrophages (TAMs). The protumorigenic TAMs are involved in critical features of neoplastic cells (such as migration & metastasis), in the inflammatory tumor microenvironment, angiogenesis, survival under hypoxia, and immune evasion [60]. Although most research groups that work on PONs employ macrophages, no study addressed TAMs to our knowledge. PON2 expression is enhanced during monocyte to macrophage differentiation in a ROS-dependent manner [28], but it is uncertain how the established anti-inflammatory and antioxidative effect of PON2 could fit particularly into TAM functions. Thus, it may be worthwhile to assess PON2 levels in M1 versus M2 macrophages. One may speculate that TAMs have low levels of PON2, which would favor ROS formation and inflammatory responses [25, 38] and may also increase production of the metastasis-augmenting IL-1*β* (at least, the latter has been shown for PON2 knockdown in endothelial cells [61]). Alternatively, given that TAMs represent an interesting therapeutic target [60], monitoring their recruitment in tumors of wild-type mice compared to those deficient in PON2 or PON3 mice may also uncover new aspects of both paraoxonases and TAM infiltration with potential therapeutic implications. A direct role of PON3 in TAMs may be unlikely considering that PON3 appeared undetectable in human macrophages [27].

## 5. Future Perspectives

### 5.1. Manipulation of PON Expression: A Double-Edged Sword?

The relevance of PON2 and PON3 to the cancer field has been demonstrated only recently. Few studies addressed their direct role, and our current knowledge appears somewhat fragmented. However, much of their antiapoptotic mechanism has been revealed. This allows tentative evaluation of their pharmacological usefulness. We presented multiple lines of evidence demonstrating that PON2 and PON3 are frequently found upregulated in cancer samples. Specific regulatory mechanisms are mostly unknown. The altered expression appears similar but also distinct for each of the two enzymes, varying with the tissue itself, with the specific kind of tumor and its stage, progression, differentiation, and/or metastasizing potency (see above). Up to now, two studies directly analyzed PON2 and PON3 levels in a variety of different tumors or representative cell lines [23, 24]. Addressing this question from the opposite direction, many independent laboratories used microarrays to investigate gene expressions in different tumors and often (though not always) found enhanced levels of these enzymes (see above).

Our current understanding allows to conclude that overexpression of PON2/PON3 diminishes the execution of the apoptotic program. Most likely, the antioxidative function of these enzymes represents the antiapoptotic trigger, whereas the contribution of their enzymatic activities remains unknown, if at all significant. How this works in the ER and relates to the UPR that is unknown. In the simplest model, however, this refers to electron transport within the inner mitochondrial membrane, which is in close proximity to and control of powerful apoptotic modifiers. From such perspective, upregulation of PON2 or PON3 in cells destined for apoptotic evasion appears consequential. As a logical deduction, the controlled reduction of overexpressed PON2 and/or PON3 in a given tumor may represent a novel approach to enhance its susceptibility to chemotherapeutics and to improve the therapy's effectiveness. Such hypothesis is encouraged by the observation that PON2 knockdown induced spontaneous apoptosis of several human tumor cell lines and because overexpression of either PON2 or PON3 granted robust chemotherapeutic resistance [23, 24]. PON2/PON3 expression varied substantially between different cell types, and high levels did not automatically correlate with cellular responsiveness to its knockdown. This outlines that individual approaches must be identified because PON2 and PON3, similar to already established targets, are unlikely to be beneficial in every setting. Therefore, future studies need to identify a rapid, reliable, and simple read-out system to monitor if a given tumor relies on high PON levels. This should be worthwhile, for example, in leukemic transformation in pediatric B-precursor ALL, where PON2 was among a very small group of factors highly expressed in patients with worst outcome, high risk, and affected relapse-free survival [18]. Other rewarding projects may be deduced from Table 1, where we summarized the combination (if available) of studies reporting PON2 overexpression in a given tumor. There is limited evidence for PON3 since we are just beginning to understand its function and because PON3, compared to PON2, is expressed to much lower levels and in fewer tissues. Given our recent data [24], we nevertheless conclude PON3 also represents a molecule actively involved in cell death regulation.

Can we then simply strive for a systemic downregulation of PON2/PON3 in selected cancer therapies and if so, which specific risks could be expected? PON2-deficient mice were in a pronounced inflammatory status [25] and suffered from a series of other defects linked to severe malfunctions (Witte & Horke; unpublished). Furthermore, (i) reduced PON2 levels enhanced atherogenesis in mice [25, 38], modulated monocyte chemotaxis and cell-mediated LDL oxidation [25, 32], and correlated with atherosclerosis progression in humans [62]; (ii) PON2 may have a neuroprotective role [63]; (iii) genetic associations linked PON2 with amyotrophic lateral sclerosis [64], Alzheimer disease [65], microvascular complications in diabetes [66], coronary heart disease [67], or perhaps also obesity [68]; (iv) PON2 plays a dominant role in the hydrolysis of bacterial virulence regulators [69–71] such that its knockdown may favor certain infections. In a similar manner, human PON3 also has a protective role against atherosclerosis and obesity [1, 33–35], but interpretation is complicated by the fact that there are conflicting reports on its expression pattern, which also varies with the species. Marsillach et al. found PON3 by immunohistochemistry in human aortic walls and macrophages [72], while we did not detect human PON3 message or protein in immortalized EA.hy 926 macrovascular endothelial cells, in primary HUVECs (human umbilical vein endothelial cells), SMCs (human coronary artery smooth muscle cells), or AoAFs (aortic adventitial fibroblasts; [24] and data not shown); this makes it difficult to reveal the mechanistic site of action. Moreover, human PON3 is present on HDL particles and absent in macrophages while the opposite is true for mice [1, 27]. In general, it has been postulated that human PON3 exerts its (antiatherogenic) function rather inside than outside the cells [35, 73], similar to PON2 and likely different from PON1. Studies performed by Shih et al. also revealed a role for PON3 in lipid metabolism that links to adiposity; intriguingly, this was gender-specific for yet unknown reasons [35]. Collectively, PON2 and PON3 have protective functions in cardiovascular diseases, and PON2 plays a dominant role in antibacterial defense, such that an untargeted knockdown may favor these illnesses. As a consequence, a systemic downregulation of PON2 or PON3 does not seem advantageous, as it likely causes a range of serious side effects.

Given that PON2/PON3 protect against atherosclerosis and stabilize atherosclerotic plaques (see above and [33, 36, 74]), may their upregulation then be beneficial to combat atherosclerosis? This is a relevant aspect given the overall number of deaths caused by cardiovascular diseases, which outnumber all cancers [75]. We would first need to determine if enforced PON2/PON3 expression blocks progression or, under optimal conditions, causes regression of established atherosclerotic plaques. Overexpression has been shown to prevent atherogenesis in murine models [25, 33, 35, 36, 74]; however, in clinical reality, patients show up with fully established plaques and need alleviating care, as it is too late for prophylactic approaches. Yet, there is little [33] or no evidence if PONs block progression of established plaques or even cause regression, perhaps due to their anti-inflammatory effects. Should such studies be positive, how can we exploit the beneficial effects of PON2/PON3 against atherosclerosis while concomitantly avoiding their pro-oncogenic function? The first step is the identification of pathways regulating PON expression and the identification of lead substances increasing or decreasing endogenous levels. Then, one solution may come from drug-eluting stents implanted into the atherosclerotic vascular wall—an already established clinical application. This would allow an upregulation of PON2/PON3 directly in the diseased vessel without promoting tumor formation in distant organs. Another solution may come from the specific targeting of effector molecules or pathways (once they are identified) for example, via surface receptors—a likely realistic mission given the accessibility of the vascular wall. In turn, similar approaches could be useful to downregulate PONs in cancer tissues. It would also be valuable to inhibit the interaction of PONs with coQ10 as this could block their antioxidative effect and render these enzymes useless for cancer cells. Finally, the time line may be advantageous in consideration of slow-progressing atherosclerosis and fast-progressing tumors; in some cases this may allow transient downregulation of paraoxonases to boost efficacy of anticancer therapies while not immediately causing plaque formation.

In summary, there exists a remarkable twist in the paraoxonase field since we know that PON2 and PON3 protect against cardiovascular diseases but favor tumor formation. It will be exciting to await further developments and the usefulness of these enzymes in the fight against two of the most significant human diseases.

## Figures and Tables

**Table 1:** Expression levels of PON2 in various tumor tissues and/or cancer cell cultures. Microarray experiment (array express) listings are according to the Gene Atlas Database. Protein and cDNA levels according to [23]. Cell culture expression levels were roughly estimated as relative level comparing to A549, grouped into *low, medium, *or *high*.

Tissue (cancer)	Protein level (fold of normal tissue)	cDNA array (fold of normal tissue)	Microarray studies	Cell culture (expression level in cell line)
Kidney	2	2.2	Upregulated in renal carcinoma (E-MTAB-37)	Medium (HEK293)
Liver	1.7	2.2	Overexpressed (Li et al. [15])	High (Huh7/HepG2)
Lung	1.3	1	Upregulated in lung adenocarcinoma (E-MEXP-231/E-MTAB-37) Downregulated in small cell lung carcinoma (E-GEOD-4127)	High (A549; H661; H1299)
Spleen	0.5	n/a		
Pancreas	1.4	1.6	Upregulated in pancreatic carcinoma (E-MTAB-37)	
Thymus	11.5	n/a		
Urinary bladder	n/a	4.1		High (HT1367/RT112)
Esophagus	n/a	0.6	Upregulated in esophageal cancer (E-MTAB-62)	
Stomach	n/a	1	Upregulated in gastric carcinoma (E-GEOD-2685)	
Ovar	n/a	1	Upregulated (E-MTAB-62)	
Cervix	n/a	1	Upregulated (E-MTAB-37/E-MTAB-62)	Medium (HeLa)
Adrenal gland	n/a	1	Downregulated in adrenocortical carcinoma (E-TABM-311)	
Thyroid gland	n/a	1.4	Upregulated (E-GEOD-3467/E-GEOD-3678)	
Prostate	n/a	1.6	Overexpressed (Ribarska, T. et al. [16] E-MTAB-62)	
Testis	n/a	1.7		Low (SuSa/GCT27/833K)
Uterus/endometrium	n/a	2.1		
Lymphoid tissue	n/a	2.5		
Leukemias (various)	n/a		Upregulated in pediatric ALL (Ross et al. and Kang et al. [17, 18])	Low in AML-like Nalm6/EOL; Jurkat Tcells; PML-like HL60/HCW2; CML-like KCL Medium in blast crisis line K562; CML-like lama; AML-like THP1/MonoMac6/HEL
Non-Hodgkin	11.9	n/a	Downregulated (E-MTAB-37)	

**Table 2:** Expression levels of PON3 in various tumor tissues and/or cancer cell cultures. Microarray experiment (array express) listings are according to the Gene Atlas Database. cDNA levels according to [24]. Cell culture expression levels were roughly estimated as relative level comparing to A549, grouped into *low, medium, *or *high*.

Tissue (cancer)	cDNA array (fold of normal tissue)	Microarray studies	Cell culture (expression level in cell line)
Kidney	2.2	Downregulated in clear cell sarcoma of the kidney (E-GEOD-2712/E-TABM-282)	Not detectable (HEK293)
Liver	4.9	Downregulated in hepatocellular carcinoma (HCC) (Choi et al. [21])	High (Huh7) Medium (HepG2)
Lung	3.4	Upregulated in lung adenocarcinoma (E-MTAB-37/E-MTAB-62)	Medium (A549)
Pancreas	3.2	Upregulated in pancreatic carcinoma (E-MTAB-37)	
Urinary bladder	3.8		Not detectable (HT1367/RT112)
Esophagus	1.8		
Stomach	9.5		
Ovar	2.1	Downregulated in ovarian serous papillary carcinomas (OSPCs) (Santin et al. [22])	
Cervix	0.5	Downregulated in cervical carcinoma (E-MTAB-62) Upregulated in cervical carcinoma (E-MTAB-37)	Not detectable (HeLa)
Adrenal gland	1.5		
Thyroid gland	2.6	Downregulated in papillary thyroid carcinoma (E-GEOD-3467)	
Prostate	4.5	Downregulated in prostate carcinoma (E-MTAB-62)	
Testis	5.3		Not detectable (SuSa/GCT27/833K)
Uterus/endometrium	16.2		
Lymphoid tissue	2.3		
Leukemias (various)	n/a		Not detectable in AML-like Nalm6; Jurkat Tcells; PML-like HL60/HCW2 blast crisis line K562; CML-like lama; AML-like THP1 high in CML-like KCL
Non-Hodgkin	n/a	Downregulated (E-MTAB-37)	

## Abbreviations

ALL: Acute lymphoblastic leukemia
CHOP: C/EBP homologous protein (growth arrest/DNA-damage inducible gene 153, GADD153)
CML: Chronic myeloid leukemia
ER: Endoplasmic reticulum
HDL: High-density lipoprotein particle
LDL: Low-density lipoprotein particle
PON: Paraoxonase
ROS: Reactive oxygen species
SOD: Superoxide dismutase
TNF-*α*: Tumor necrosis factor-*α*
TRAIL: Tumor necrosis factor-related apoptosis inducing ligand
UPR: Unfolded protein response.
